# Atypical Case of Pemphigus Erythematosus in a Previously Healthy Young Woman: Diagnostic Delay and Response to Rituximab

**DOI:** 10.7759/cureus.97108

**Published:** 2025-11-17

**Authors:** Nadia Kirmani, Evan Shegog

**Affiliations:** 1 Medicine, School of Medicine, Stanford University, Stanford, USA; 2 Medicine, Stanford University Hospital, Stanford, USA

**Keywords:** an unusual type of pemphigus, biologics agents, pemphigus, rituximab therapy, senear-usher syndrome

## Abstract

Pemphigus erythematosus, also known as Senear-Usher syndrome, is a rare localized variant of pemphigus foliaceus characterized by superficial blistering in seborrheic regions and immunopathologic overlap with systemic lupus erythematosus. This report describes an otherwise healthy 33-year-old woman with no significant past medical history presenting with widespread erosive plaques and crusted lesions predominantly in seborrheic areas, positive desmoglein-1 antibodies, positive antinuclear antibody (ANA), and histopathology consistent with intraepidermal acantholysis. Our patient demonstrated clinical improvement after systemic corticosteroids and rituximab, with no new bullae formation. This case highlights the diagnostic challenges associated with pemphigus erythematosus, the importance of clinicopathologic correlation, and the potential role of biologic therapy in the treatment of this condition.

## Introduction

Pemphigus is a rare group of autoimmune blistering disorders characterized by IgG autoantibodies directed against intercellular adhesion proteins, leading to loss of keratinocyte adhesion and formation of intraepidermal blisters [1,2]. The major subtypes include pemphigus vulgaris, pemphigus foliaceus, immunoglobulin A (IgA) pemphigus, and paraneoplastic pemphigus.

Pemphigus foliaceus (PF) is a superficial form of pemphigus that presents with cutaneous lesions without mucosal involvement [3]. PF appears as scattered superficial blisters that develop into crusted erosions on an erythematous base and typically involve the scalp, face, and upper trunk [4]. This disease is mediated by autoantibodies against desmoglein-1, a desmosomal cadherin found in the upper epidermis [5]. Histopathology demonstrates acantholysis in the granular layer, and direct immunofluorescence (DIF) reveals intercellular deposition of IgG and complement in a “chicken wire” pattern [6].

Pemphigus erythematosus (Senear-Usher syndrome) is categorized as a localized variant of PF. Senear-Usher syndrome presents with superficial erosions and crusts in seborrheic regions such as the scalp, cheeks, and upper trunk, and may demonstrate immunologic features associated with systemic lupus erythematosus, such as positive ANA or a lupus band pattern on DIF [7]. However, patients often do not fulfill systemic lupus erythematosus (SLE) criteria because they typically lack systemic features such as renal, hematologic, joint, or serosal involvement [8].

Epidemiologically, pemphigus erythematosus is rare, accounting for a small fraction of pemphigus cases. It is more prevalent in endemic regions of North Africa and South America, affecting middle-aged adults, with a slight female predominance; however, cases in younger individuals have been reported less frequently [9].

Seborrheic pemphigus is another localized form of PF presenting in seborrheic areas and has historically been confused with pemphigus erythematosus; differentiation requires careful evaluation of clinical distribution, histopathology, immunofluorescence findings, and systemic features [10]. Here, we present a case of pemphigus erythematosus in a young woman with no autoimmune history, highlighting diagnostic challenges and clinical response to systemic corticosteroids and rituximab.

## Case presentation

A 33-year-old woman with no significant past medical history presented with a one-year history of intermittent blistering involving the face, trunk, and extremities, with significant worsening over the preceding four weeks. She first sought care at an outside hospital, where lesions were suspected to represent impetiginized dermatitis or cellulitis. She was treated empirically with hydroxyzine, low-dose topical hydrocortisone, trimethoprim-sulfamethoxazole, and cephalexin, but showed no improvement.

**Figure 1:**
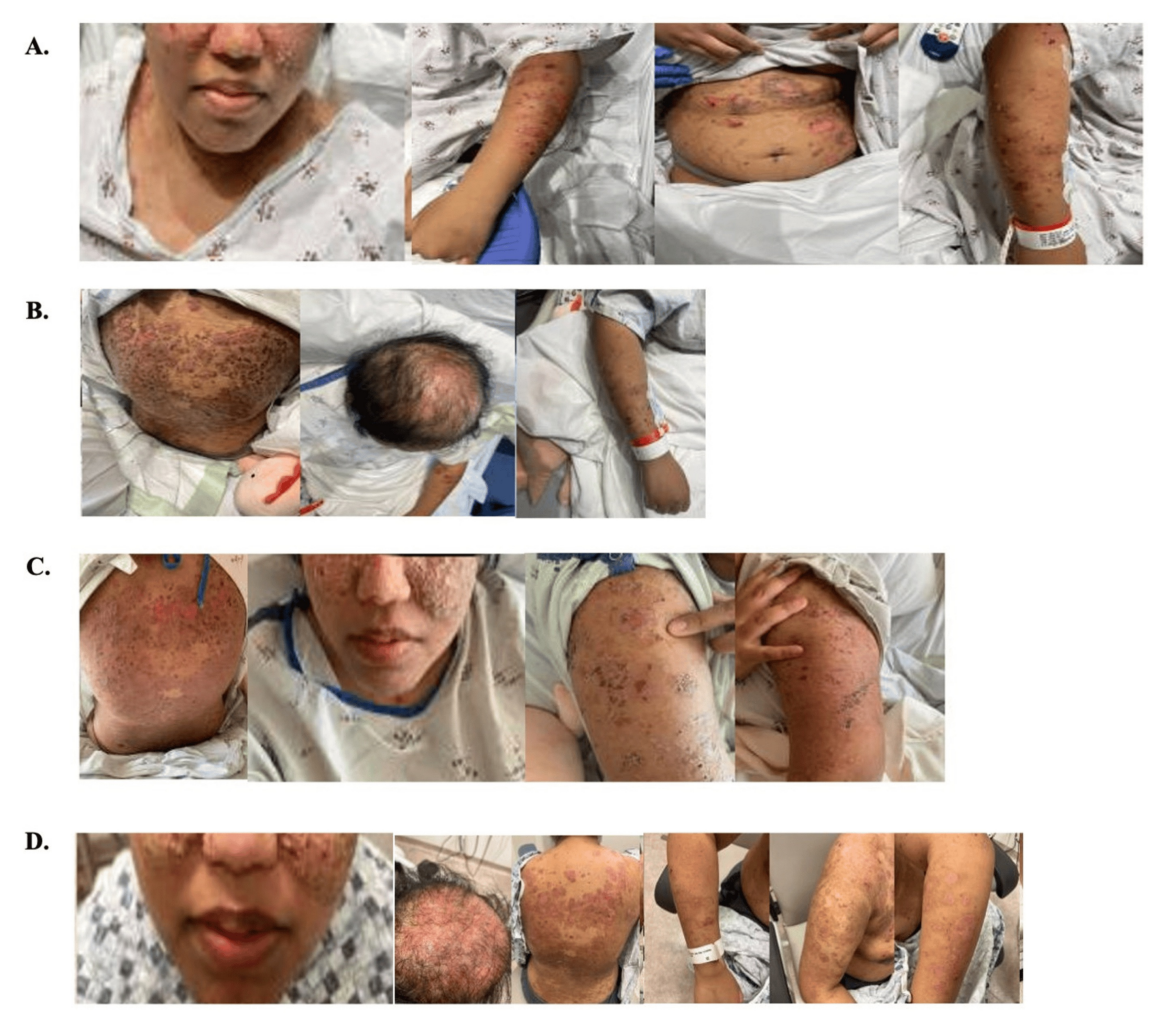
Skin manifestations of pemphigus erythematosus in a 33-year-old female. The images show (A) the initial presentation, (B) after IV Solu-Medrol and the first dose of rituximab (1 g), (C) after the prednisone taper, and (D) on the day of discharge.

She presented to our institution four weeks later due to progression of her symptoms and development of widespread painful erosions. On admission, examination demonstrated superficial erosions, crusted plaques, and flaccid bullae across the cheeks, nose, chest, abdomen, upper extremities, and back. Lesions were erythematous and exudative, exhibiting a positive Nikolsky sign. There was no involvement of the oral, ocular, or genital mucosa (Figure 1, panel A).

Laboratory evaluation revealed a positive ANA (1:320, homogeneous pattern). A punch biopsy of the right abdomen demonstrated intraepidermal acantholysis with “tombstoning” of basal keratinocytes. Serologies for Scl-70, Ro52, Ro60, and La antibodies were negative. Autoantibodies against desmoglein-1 were markedly elevated, while desmoglein-3 antibodies were negative.

The patient was treated with intravenous methylprednisolone 125 mg daily for three days and received 1 g of rituximab on hospital day one. Within 72 h, no new bullae formed, and existing lesions showed reduced erythema and pain (Figure 1, panel B). She was transitioned to oral prednisone 100 mg daily with a taper plan (Figure 1, panels C, D).

At outpatient follow-up, the patient reported overall improvement but noted a few new superficial erosions on the forearms and cheeks. Examination demonstrated healing erosions with overlying hemorrhagic or honey-colored crusts on the trunk and extremities. A second perilesional biopsy for direct immunofluorescence (DIF) was performed at this visit, after initiation of steroids and rituximab, and demonstrated intercellular deposition of IgG and complement C3. The patient subsequently received a second 1 g dose of rituximab. She continued to improve clinically with a reduction in lesion frequency, pain, and fatigue.

## Discussion

Pemphigus erythematosus (Senear-Usher syndrome) is a rare variant of pemphigus foliaceus characterized by superficial blistering in seborrheic regions and serologic overlap with systemic lupus erythematosus. Diagnosis requires correlation of clinical features, histopathology, serologic testing, and immunofluorescence findings. In this case, widespread erosions, elevated desmoglein-1 antibodies, positive ANA, and intraepidermal acantholysis on biopsy supported the diagnosis of pemphigus erythematosus.

A major diagnostic limitation in this case was the timing of direct immunofluorescence (DIF), which was performed only after initiation of systemic therapy. DIF is ideally obtained from perilesional skin before corticosteroids or immunosuppressive agents are started, as these therapies can decrease immunoreactant deposition and reduce diagnostic sensitivity [11].

The differential diagnosis included seborrheic pemphigus, paraneoplastic pemphigus, and bullous systemic lupus erythematosus (SLE). Seborrheic pemphigus typically presents with localized lesions that respond to topical therapies, whereas this patient demonstrated widespread involvement and positive desmoglein-1 antibodies, which are more consistent with pemphigus erythematosus. Paraneoplastic pemphigus was unlikely due to the absence of mucosal involvement, desmoglein-3 antibodies, polymorphic lesions, or underlying malignancy. Bullous SLE was considered, given the high-titer ANA; however, the patient lacked systemic features, and further lupus serologies, including anti-dsDNA, anti-Smith, anti-RNP, and complement levels, were within normal limits.

Rituximab, an anti-CD20 monoclonal antibody, has shown efficacy in pemphigus diseases, particularly in steroid-refractory or severe cases [12]. Clinical trials in pemphigus vulgaris have demonstrated superior remission rates and reduced corticosteroid requirements compared to corticosteroids alone [13]. Although data specific to pemphigus erythematosus remain limited to case reports and small series, this case supports the potential role of rituximab as an effective adjunct to systemic corticosteroids. Decisions regarding maintenance dosing remain individualized and may depend on clinical response, B-cell repopulation, and serial autoantibody levels.

This case is limited by the lack of histopathology images and delayed DIF testing, which may reduce diagnostic certainty. Nevertheless, it contributes to existing literature by highlighting an atypical presentation of pemphigus erythematosus in a young woman outside of commonly reported demographics and illustrates the therapeutic potential of rituximab in disease management.

## Conclusions

This case highlights an atypical presentation of pemphigus erythematosus in a young female without prior autoimmune disease. Diagnosis required correlating clinical findings with biopsy, serology, and direct immunofluorescence. Early initiation of systemic corticosteroids and rituximab led to clinical improvement. While this case supports the use of biologic therapy in pemphigus erythematosus, further research is needed to establish long-term efficacy, optimal dosing, and maintenance strategies. Prompt biopsy with both histopathology and DIF before immunosuppressive treatment is critical for accurate diagnosis.

## References

[1] Mihai S, Sitaru C (2007). Immunopathology and molecular diagnosis of autoimmune bullous diseases. J Cell Mol Med.

[2] Sitaru C, Zillikens D (2005). Mechanisms of blister induction by autoantibodies. Exp Dermatol.

[3] Meyer N, Misery L (2010). Geoepidemiologic considerations of auto-immune pemphigus. Autoimmun Rev.

[4] Defendi F, Thielens NM, Clavarino G, Cesbron JY, Dumestre-Pérard C (2020). The immunopathology of complement proteins and innate immunity in autoimmune disease. Clin Rev Allergy Immunol.

[5] Kershenovich R, Hodak E, Mimouni D (2014). Diagnosis and classification of pemphigus and bullous pemphigoid. Autoimmun Rev.

[6] Kridin K (2018). Pemphigus group: overview, epidemiology, mortality, and comorbidities. Immunol Res.

[7] Uzun S, Durdu M, Akman A, Gunasti S, Uslular C, Memisoglu HR, Alpsoy E (2006). Pemphigus in the Mediterranean region of Turkey: a study of 148 cases. Int J Dermatol.

[8] Hagihara E, Ito H, Okamoto T, Inomata N (2023). A case of apple-dependent, exercise-induced anaphylaxis with sensitization to gibberellin-regulated proteins. J Dermatol.

[9] Kutwin M, Kądziela M, Stein T, Kraska-Gacka M, Woźniacka A, Żebrowska A (2025). Senear-Usher syndrome or coexistence of SLE with pemphigus vulgaris - a case report with literature review. J Clin Med.

[10] Didona D, Di Zenzo G (2018). Humoral epitope spreading in autoimmune bullous diseases. Front Immunol.

[11] Lehman JS, Johnson EF, Camilleri MJ (2022). Impact of adding an IgG4 conjugate to routine direct immunofluorescence testing for subepithelial and intraepithelial autoimmune blistering disorders. J Cutan Pathol.

[12] Kaegi C, Wuest B, Schreiner J (2019). Systematic review of safety and efficacy of rituximab in treating immune-mediated disorders. Front Immunol.

[13] Joly P, Horvath B, Patsatsi Α (2020). Updated S2K guidelines on the management of pemphigus vulgaris and foliaceus initiated by the European Academy of Dermatology and Venereology (EADV). J Eur Acad Dermatol Venereol.

